# Laparoscopic partial splenectomy for giant cyst using a radiofrequency-assisted device: a case report

**DOI:** 10.1186/s40792-016-0206-x

**Published:** 2016-08-24

**Authors:** R. Quesada, I. Poves, M. Iglesias, E. Berjano, L. Grande, F. Burdío

**Affiliations:** 1 Department of Experimental and Health Sciences, Universitat Pompeu Fabra, Parc de Recerca Biomèdica de Barcelona Doctor Aiguader 88, Barcelona, 08003 Spain; 2 Unit of Hepato-Biliary and Pancreatic Surgery, Department of Surgery, Hospital del Mar, Barcelona, Spain; 3 Department of Pathology, Hospital del Mar, Barcelona, Spain; 4 Biomedical Synergy, Electronic Engineering Department, Universitat Politècnica de València, Valencia, Spain

**Keywords:** Giant splenic cyst, Laparoscopic splenectomy, Radiofrequency

## Abstract

**Background:**

Although radiofrequency-assisted devices have sometimes been used in partial splenectomy, this is not a common technique. This report describes the first case of laparoscopic partial splenectomy using an RF-assisted device (Coolinside) which allows both coagulation and transection of the parenchyma and eventually the protective coagulation of the remnant side.

**Case presentation:**

A 27-year-old woman was found to have a giant hydatic cyst measuring 12.0 × 14.0 × 16.6 cm that mainly occupied the lower pole of the spleen and retroperitoneal space. The patient underwent a laparoscopic partial splenectomy using an RF-based device designed to accomplish both the coagulation and dissection of the splenic tissue. The estimated blood loss was less than 200 mL.

**Conclusions:**

Even though RF ablation has traditionally been used for hepatic parenchymal transection, it seems equally suited to partial splenectomy. This device seems to provide good results, minimizing blood loss during partial splenectomy; however, randomized trials will be necessary to see if the results are superior to those of other techniques.

## Background

Partial splenectomy is increasingly being advocated as the standard of care for symptomatic splenic cysts [1]. Unfortunately, as achieving adequate hemostasis during partial splenectomy is still a great challenge, the use of the technique is currently limited. In the past, our group developed a pencil-type sealer-dissector electrosurgical device intended to minimize blood loss during surgical resection [2], based on an internally cooled radiofrequency (RF) electrode with a built-in blade for easy dissection of the coagulated tissue to avoid using an additional device. This feature has been shown to be valuable in laparoscopic surgery. We have now gained broad experience in using this device in laparoscopic hepatic surgery, which has encouraged us to expand its use towards other organs. The device is currently commercialized as the Coolinside Device (Apeiron Medical, Valencia, Spain). In the present case study, this device was utilized to reduce blood loss in a partial laparoscopic splenectomy for a symptomatic traumatic cyst.

## Case presentation

**Fig. 1:**
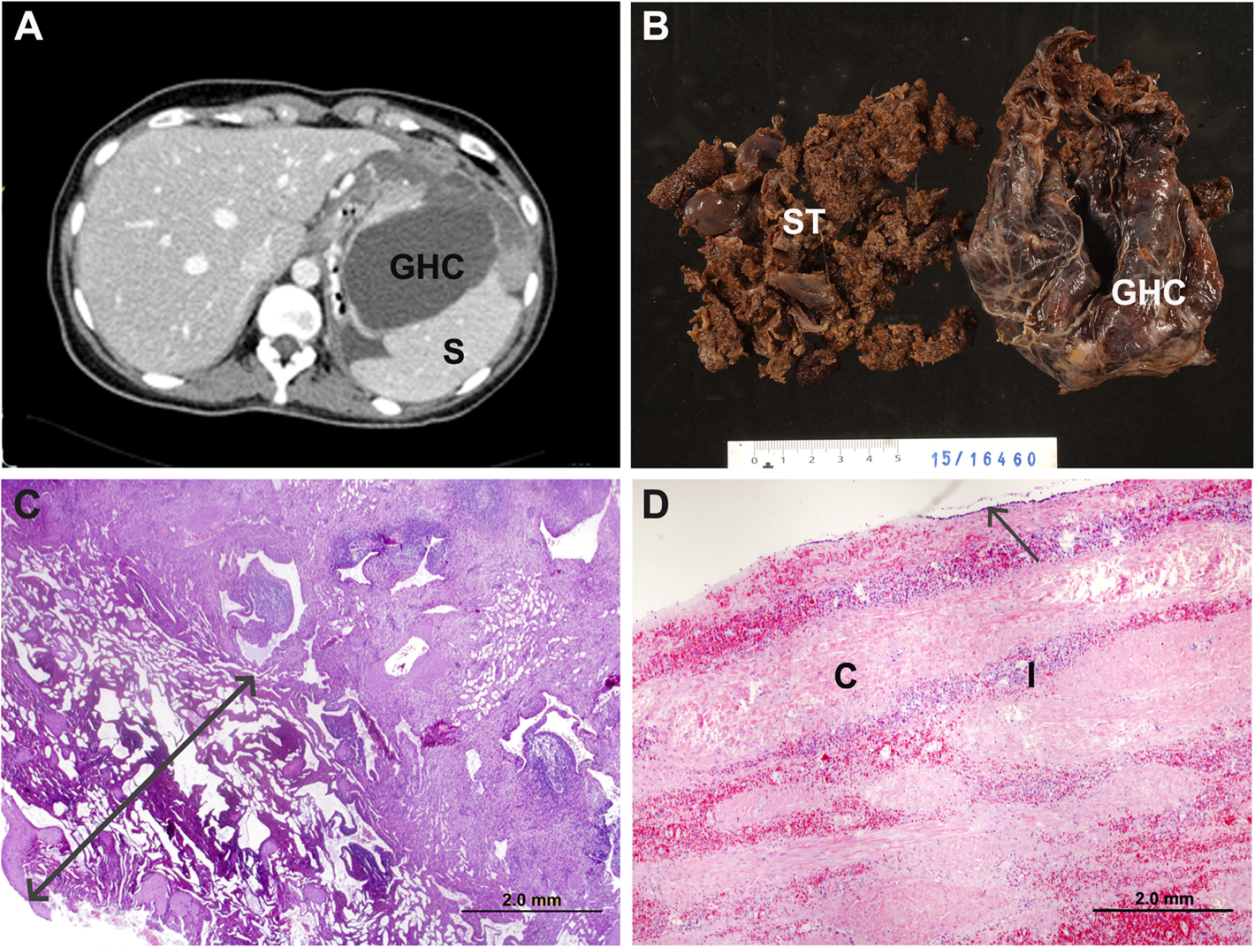
**a** Preoperative CT image of the spleen showing the giant hydatic cyst (*GHC*) occupying the lower pole of the spleen (*S*). **b** Macroscopic piece of the GHC with contiguous spleen tissue (*ST*) after partial splenectomy. **c** Histological image of the margin of transection (the *arrow* indicates the width of the thermally coagulated margin). **d** Histological image of the typical structure of the GHC showing the cuboidal epithelium (arrow), a layer of connective tissue (*C*) followed by a congestive area with slight inflammatory components (*I*)

A 27-year-old woman suffering from mild epigastralgia and constipation was admitted to the hospital. On clinical examination, an enlarged tender abdominal mass was palpable in the left upper quadrant 6-cm below the costal margin. Thrombocytopenia and leucopenia were also detected. Abdominal ultrasound and CT scan revealed a 19 cm splenomegaly nearby completely occupied by a giant cyst measuring 12.0 × 14.0 × 16.6 cm (approximate volume 1400 cm^3^) and another smaller 1.5 cm cyst. These cysts occupied the greater part of the lower pole and retroperitoneal space (see Fig. 1a). The white blood cell (WBC) count was 3.1 × 10^3^ cells/μL, hemoglobin was 127 g/L, MCV was 80 fL and platelet count was 109 × 10^3^/μL.

**Fig. 2:**
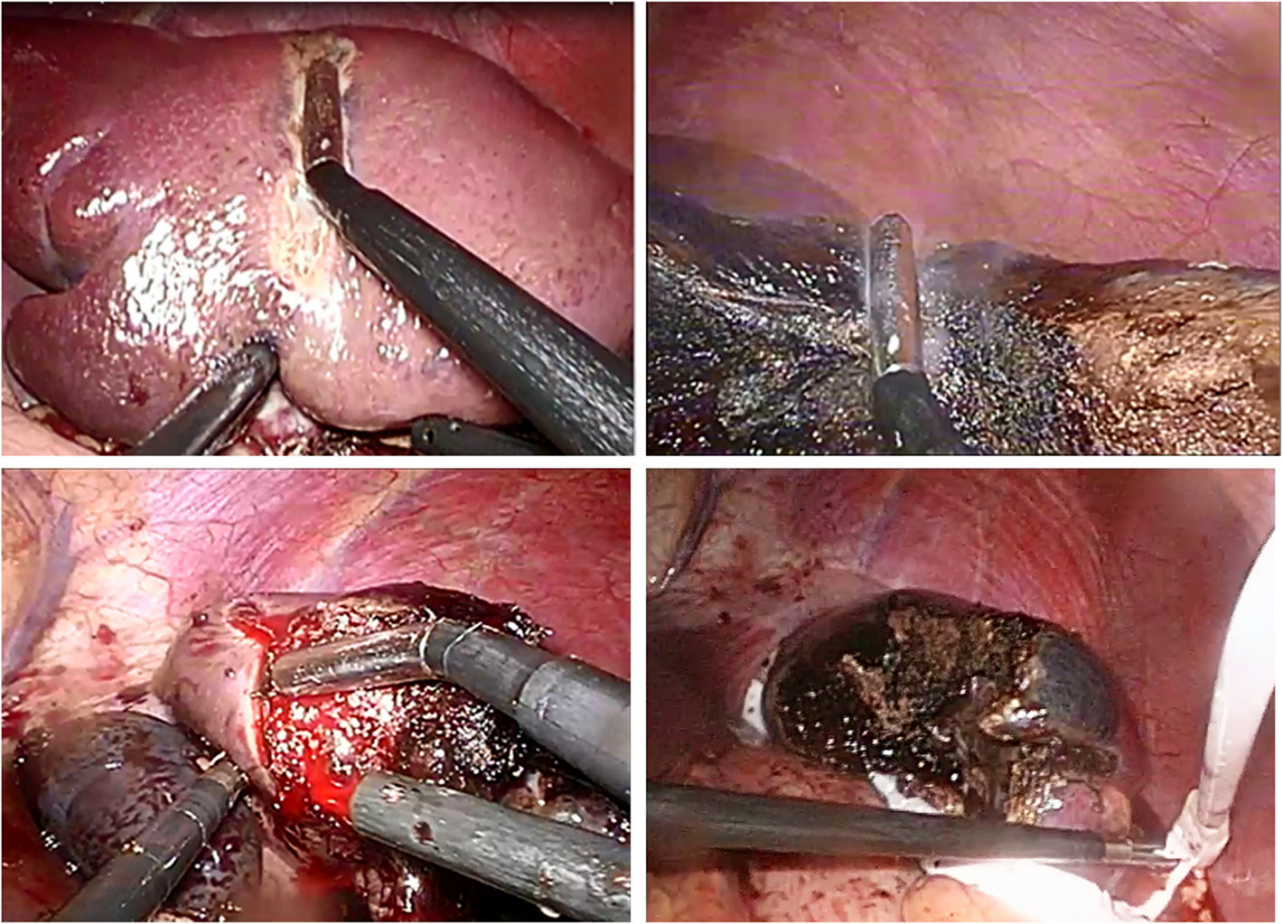
Images of the Coolinside device running along the transection line of the partial splenectomy. Finally, there is the preserved pole of the spleen and bloodless cut surface

Before surgery, a prophylactic treatment of amoxicillin was prescribed. A 12-mm camera port was placed in the periumbilical position under an open technique to create penumoperitoneum. Three (one 10 mm and two 5 mm) working ports were placed to the left and right of the camera port. First, the spleen was fully mobilized by dividing the splenocolic and gastrosplenic ligaments. Short gastric vessels were divided with the Ligasure device (Valleylab, Boulder, CO, USA), and a giant splenic cyst was found. A total of 1800 cm^3^ of fluid was aspirated. Dissection and coagulation of the spleen parenchyma was achieved by using a Ligasure device (Valleylab, Boulder, CO, USA) and Coolinside Device Model 5L11 (Apeiron Medical, Valencia, Spain), respectively. The partial splenectomy was performed resecting the vast majority of the cyst leaving a small portion of the capsule in the remnant spleen. The Coolinside device was connected to the Apeipower RF generator and a power level of 9 (equivalent to 200 W) was set (Fig. 2). Four dispersive pads (118 cm^2^ each) were placed on the patient’s back and thighs. No vascular clamping was necessary. The estimated blood loss was less than 200 mL. No unusual events were observed during the postoperative period. The patient was discharged 3 days after surgery.

The histological analysis showed splenic parenchyma with normal architecture without evidence of a lymphoproliferative process. Figure 1b shows a macroscopic view of the giant hydatic cyst with contiguous spleen tissue. The histological analysis revealed a ~5 mm deep transection margin (thermally coagulated zone) (Fig. 1c). We also observed an area of cystic aspect lined by epithelium with cuboidal cells (see Fig. 1d) and dystrophic calcification areas, which is the typical lining of an epithelial (primary) splenic cyst.

Nine days after discharge, the patient was readmitted due to pain in the left side of the abdomen for 24 h without nausea or vomiting or changes in bowel or urinary habits. The abdominal echography revealed normal changes in the splenic morphology associated with the partial splenectomy and an evident fluid serous collection. Taking the clinical findings into account, the patient was readmitted. Four days after the readmission, a pigtail catheter was used to drain 340 mL of fluid. The culture results were negative, ruling out abscess and hematoma. The patient was discharged a day later (5 days after readmission). Eight months after surgery, the patient was asymptomatic and the platelet count did not show changes (platelet count was 109 × 10^3^/μL).

### Discussion

RF energy has been shown to be a safe and effective method of thermal coagulation of tissue, both in splenic preservation surgery after traumatic splenic rupture [3–6] and during surgical splenectomy. The first surgical cases were conducted via laparotomy and used either single RF electrodes or RF devices based on needle-arrays [1, 7–9]. The laparoscopic approach has only been conducted in a few cases [10–12]. Using needle-like devices means that the dissection of the splenic parenchyma has to be conducted with an additional device (e.g., surgical scalpel or an ultrasonic dissector [10]) through the midline of the previously coagulated plane. Prior to this, the electrodes have to be carefully inserted into the tissue and an appropriate protocol for delivering RF power has to be set in order to create a 1-cm thick coagulated parenchyma resection plane. These protocols often involve replacement of the electrodes, and hence several RF application runs (up to 14) which make the procedure longer, with an RF phase up to 50 min long [12]. Unlike the needle-like devices, the saline-linked device (TissueLink Medical Inc, Dover, NH, USA) is a pencil-type electrosurgical device based on an externally irrigated monopolar RF electrode. The surgeon moves it continuously over the tissue surface to be coagulated. There are only three studies reporting the use of this device for partial splenectomy [13–15]. Although some design changes have been implemented in this device to make dissection feasible, transection of the coagulated tissue is usually accomplished with a second device, such as scissors [14] or ultrasonic dissector [13]. This report describes the first case of laparoscopic partial splenectomy using the Coolinside device, which provides both coagulation and transection of the parenchyma and eventually the protective coagulation of the remnant side. The device seems to provide excellent results in minimizing intraoperative and postoperative blood loss without clamping maneuvers. In contrast to previous techniques described [7], this device facilitates the recognition of structures during the transection of the spleen, creating a wide band of coagulated tissue adjacent to the line of transection. In the case of liver resection, the band may reach a distance of up to 1 cm from the resection margin, as found in previous experimental and clinical studies [2, 16, 17]. In the spleen, the coagulated tissue was up to 5 mm thick, which seems to be greater than the depth obtained with other devices [13–15], although this depth may vary with the duration of the contact and power setting. Therefore, some potential limitation should also be addressed: a complete knowledge of the consequences of leading a wide band of coagulation the remnant spleen is not yet available and this greater coagulation may lead to a smaller remnant spleen.

## Conclusions

Laparoscopic partial splenectomy using the Coolinside device appears to be safe and effective in terms of transection and hemostasis, which permits the preservation of the splenic function and may be particularly relevant in the treatment of abdominal trauma of the spleen. However, future randomized and prospective trials will be necessary to determine whether this device is superior to other techniques.

## Abbreviations

CT, computed tomography; RF, radiofrequency
